# Targeting the Heart of Mycobacterium: Advances in Anti-Tubercular Agents Disrupting Cell Wall Biosynthesis

**DOI:** 10.3390/ph18010070

**Published:** 2025-01-09

**Authors:** Ahmad Diab, Henry Dickerson, Othman Al Musaimi

**Affiliations:** 1 School of Pharmacy, Newcastle University, Newcastle upon Tyne NE1 7RU, UK; h.dickerson2@newcastle.ac.uk; 2 Department of Chemical Engineering, Imperial College London, London SW7 2AZ, UK

**Keywords:** mycobacterium tuberculosis, isoniazid, delamanid, mycolic acid, cycloserine, peptidoglycan, arabinogalactan, multi-drug resistance, clinical trials

## Abstract

*Mycobacterium tuberculosis* infections continue to pose a significant global health challenge, particularly due to the rise of multidrug-resistant strains, random mycobacterial mutations, and the complications associated with short-term antibiotic regimens. Currently, five approved drugs target cell wall biosynthesis in *Mycobacterium tuberculosis*. This review provides a comprehensive analysis of these drugs and their molecular mechanisms. Isoniazid, thioamides, and delamanid primarily disrupt mycolic acid synthesis, with recent evidence indicating that delamanid also inhibits decaprenylphosphoryl-β-D-ribose-2-epimerase, thereby impairing arabinogalactan biosynthesis. Cycloserine remains the sole approved drug that inhibits peptidoglycan synthesis, the foundational layer of the mycobacterial cell wall. Furthermore, ethambutol interferes with arabinogalactan synthesis by targeting arabinosyl transferase enzymes, particularly embB- and embC-encoded variants. Beyond these, six promising molecules currently in Phase II clinical trials are designed to target arabinan synthesis pathways, sutezolid, TBA 7371, OPC-167832, SQ109, and both benzothiazinone derivatives BTZ043 and PBTZ169, highlighting advancements in the development of cell wall-targeting therapies.

## 1. Introduction

Bacterial infections remain a global health crisis, exacerbated by the rise of multidrug-resistant strains. Currently, approximately 700,000 deaths per year are attributed to drug-resistant infections, with predictions suggesting this number could surpass 10 million deaths annually by 2050 [1]. Alexander Fleming’s understanding of antimicrobial resistance in the 1940s and 1950s has led to more cautious prescribing practices, resulting in shorter-term use of antibiotics and efforts to limit their prescription to prevent the development of resistance [1]. This modern-day prescribing phenomenon has been costly to the pharmaceutical industry, putting pressure on sales and profits. Consequently, it has led to a decreased interest in researching and developing new antibiotics [2]. Other challenges include the prolonged discovery pipeline, limiting treatment availability and options, and spontaneous bacterial mutations, which have dampened scientific innovation in developing new antibacterial agents for decades [3]. *Mycobacterium tuberculosis* (Mtb), also known as Koch’s bacillus, is a highly contagious acid-fast bacterium that causes chronic pulmonary infections. These infections can spread and lead to multiple secondary infections, including meningitis and lymphadenitis, with an increased incidence of HIV-Mtb coinfections [4]. The classification of Mtb is controversial: it has a unique hydrophobic cell containing mycolic acid (MA) which prevents the microorganism from Gram staining, and the cell wall also contains efflux pumps; therefore, it is best classified as an acid-fast bacillus [5].

Although the structure of Mtb was studied as far back as 1882 by German physician and Nobel Laureate Robert Koch, clinical concerns still persist, making Mtb infections a global health concern [6]. Clinical features of Mtb infection can be challenging to diagnose in primary care settings. The bacteria can enter a latent phase where they display no symptoms, or they can cause symptoms that mimic other pathologies, such as pneumonia. This complexity requires standardised chemotherapy protocols tailored to treat tuberculosis (TB) effectively [4]. According to the 2020 global report, TB caused 1.4 million deaths in 2019. Improved hospital infection control and the Bacille Calmette–Guérin (BCG) vaccine in some countries have reduced paediatric TB-related deaths over the years. However, TB remains among the top ten leading causes of death globally [7,8]. In addition, drug discovery challenges have resulted in only five lead compounds being approved since 1953 to directly target cell wall biosynthesis. This was the year when isoniazid (INH) was first approved, highlighting the slow pace of new drug approvals in this specific area of TB treatment (Table 1) [9].

The composition of the cell wall and the presence of efflux pumps on the Mtb cell wall present challenges in drug discovery due to the wall’s highly lipophilic nature. This characteristic makes it difficult for drugs to penetrate the cell wall effectively, limiting their efficacy against Mtb infections [18]. Considering the thickness and composition of the cell wall, which includes peptidoglycan (PG), arabinogalactan (AG), MA, lipoarabinomannan (LAM), phosphatidylinositol mannosides, and sulfolipids, Mtb possesses a robust physical barrier that provides high protection against foreign substances, including antimicrobial agents. This complex structure contributes significantly to the bacterium’s ability to resist and survive various environmental stresses and antimicrobial treatments [18].

Current treatment for Mtb infections typically involves a combination of antibiotics targeting multiple aspects of the bacterium’s physiology and cell wall biosynthesis. These commonly used drugs include INH, rifampicin (RIF), pyrazinamide (PZA), and ethambutol (EMB), two of which target cell wall synthesis (INH, EMB) to ensure effective treatment and minimise the development of resistance (Table 1). INH, thioamides, and delamanid disturb the cell wall biosynthesis by blocking MA synthesis [9,10,18]. This disruption is vital for halting Mtb growth and replication, as MAs are key components of the bacterial cell wall, ensuring its structural integrity and permeability [9,18]. On the other hand, cycloserine inhibits the enzymes D-alanine racemase and ligase, disrupting PG synthesis, a vital component of Mtb’s cell wall. This interference weakens the cell wall’s structural integrity, impairing the bacterium’s ability to maintain its shape and resist external stresses [9,18]. Finally, ethambutol inhibits arabinosyl transferase enzymes, blocking AG synthesis, a critical component of Mtb’s cell wall responsible for its structural integrity and permeability. This disruption compromises the cell wall’s function, impairing Mtb’s survival and replication. Ethambutol is typically prescribed for the first 2 months of treatment [18]. This approach is approved for up to 6 months to prevent the development of resistance and reduce mortality rates, particularly in elderly and HIV-infected patients who are more susceptible to severe TB infections.

The treatment plan for TB is guided by the duration and the bacteria’s resistance profile, following guidelines from organisations like the World Health Organization (WHO) and the Centres for Disease Control and Prevention (CDC). TB infections are primarily diagnosed using two strategies: the interferon gamma release assay (IGRA) blood test and the Mantoux tuberculin skin test (TST). IGRA is preferred over TST because BCG-vaccinated patients may yield false positive results with the skin test (Table 2). On the other hand, latent TB infections, which are asymptomatic during clinical evaluations, require additional diagnostic tests such as microscopy and chest radiographs to confirm the diagnosis.

Treatment for latent TB typically involves INH monotherapy for 6–9 months or a combination of INH and RIF for 3 months. Alternatively, RIF monotherapy for 4 months is recommended for patients at risk of hepatotoxicity.

Active TB treatment follows a two-step regimen: intensive and continuation phases. (i) Intensive phase (4-month regimen): includes rifapentine (RPT), moxifloxacin (MOX), INH, and pyrazinamide; (ii) 6-month regimen: MOX and RPT are replaced with EMB and RIF; (iii) continuation phase: (A) 4-month regimen: INH, MOX, and RPT, (B) 6–9-month regimen: INH and RIF.

These treatments may be adjusted based on patient comorbidities, coexisting infections like HIV, and potential drug interactions (Table 2).

IGRA, interferon gamma release assay; INH, isoniazid; MOX, moxifloxacin; PZA, pyrazinamide; RIF, rifampicin; RPT, rifapentine; TST, tuberculin skin test.

Multi-drug-resistant TB (MDR-TB) is identified when the strain exhibits resistance to both first-line therapies, INH and RIF. Extensively drug-resistant TB (XDR-TB) is diagnosed when the strain is resistant to INH, RIF, a fluoroquinolone, and at least one injectable third-line drug, such as amikacin. These classifications help tailor treatment regimens, often requiring combinations of second- and third-line antibiotics to effectively manage drug-resistant TB and reduce treatment failure risks [21].

This review explores the synthesis pathways of Mtb cell components, connecting them to approved drugs and their primary targets. It examines the chemical structures of these drugs, analysing their antitubercular activity while addressing concerns about their usage. Additionally, drugs currently in the development pipeline will be discussed.

## 2. Cell Wall Components

The initial plasma membrane of Mtb is composed of glycerol-based phospholipids, primarily phosphatidylethanolamines. Beyond the periplasm, the space between the plasma membrane and peptidoglycan (PG), lipomannan (LM), and LAM are anchored to the plasma membrane and extend through the periplasm into the outer membrane [22]. These components are characterised by their oligosaccharide structures, which are essential in the virulence and pathology of Mtb infections [18,23].

The second component is peptidoglycan (PG), found in both Gram-negative and Gram-positive bacteria. It is a polymer made up of *N*-acetylglucosamine (GlcNAc) and *N*-acetylmuramic acid (MurNAc), connected by β-1,4 glycosidic bonds. PG also includes various amino acids, resulting in structural variations among different species of Gram-positive bacteria. These polymer layers are linked by pentaglycine bridges, which help maintain the cell wall’s shape and increase its osmotic stability [18,23].

In Mtb, the PG sugar side chain is connected to AG, a complex polysaccharide. AG, in turn, branches and links to MA, a key component of the mycobacterial outer membrane. MA is highly lipophilic and plays a vital role in the bacterium’s virulence by enhancing its impermeability and preventing the fusion of phagosomes with lysosomes in host macrophages. This mechanism allows Mtb to evade the host immune response and persist within host cells [18,23].

**Figure 1:**
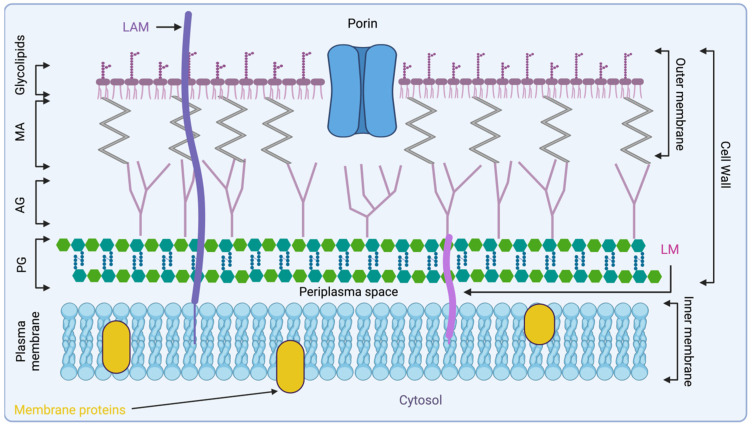
Representation of Mtb cell wall architecture, starting from the peptidoglycan polymer connected to arabinogalactan, which is connected to MA. Glycolipids are displayed on the surface of the mycobacterial cell wall with lipoarabinomannan (LAM). Created with biorender.com. AG, arabinogalactan; LAM, lipoarabinomannan; LM, lipomannan; MA, mycolic acid; PG, peptidoglycan.

The fluidity of the Mtb outer membrane is preserved by a glycolipid network mainly composed of aliphatic MAs, particularly those with C70 and C90 chain lengths. This network is further supported by other molecules, such as sulfoglycolipids, diacyltrehaloses, and phthiocerol dimycocerosates. These components are essential for maintaining the structural integrity and permeability of the outer membrane, while also contributing to the bacterium’s virulence (Figure 1) [18,23].

## 3. PG Synthesis

**Figure 2:**
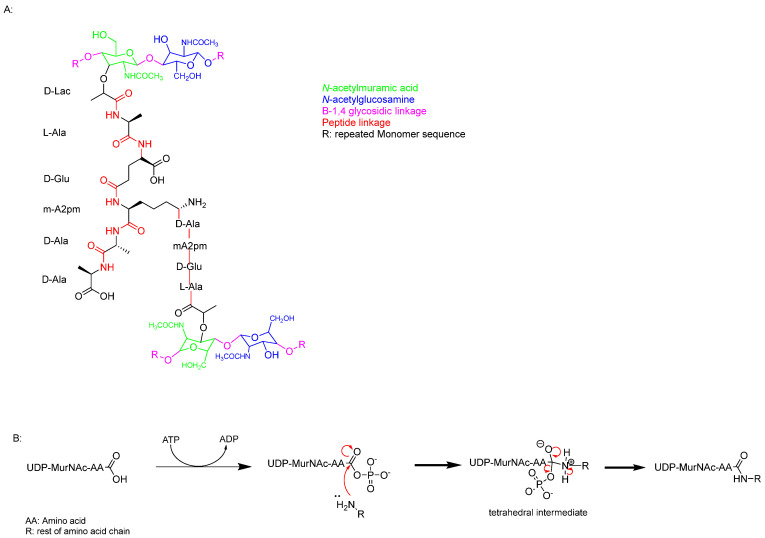
(**A**) Single monomer of the PG chain. (**B**) Nucleophilic acyl substitution mechanism linking the pentapeptide together.

PG monomer synthesis begins with the formation of uridine, (UDP)-MurNAc and UDP-GlcNAc (Figure 2). These compounds act as essential precursors for the biosynthesis of PG [24].

PG synthesis involves a series of enzyme-controlled steps, starting with the formation of UDP-GlcNAc. This compound is derived from fructose-6-phosphate through the action of GlmU, an acetyltransferase/uridyltransferase [25,26]. GlmU catalyses the conversion of glucosamine-1-phosphate to *N*-acetylglucosamine-1-phosphate, which is subsequently transformed into UDP-GlcNAc through a reaction with uridine triphosphate (UTP) [25,26,27]. Next, UDP-MurNAc is synthesised in two enzymatic steps. The first step involves MurA (UDP-N-acetylglucosamine enolpyruvyltransferase), which catalyses the transfer of an enolpyruvyl group from phosphoenolpyruvate to UDP-GlcNAc. This step is crucial for the formation of the PG precursor [28,29].

UDP-N-acetylenolpyruvyl-glucosamine reductase (MurB) is an NADPH-dependent oxidoreductase enzyme that reduces the enolpyruvate moiety of UDP-N-acetylglucosamine to form UDP-N-acetylmuramic acid (UDP-MurNAc) by converting the enolpyruvate to D-lactate. UDP-GlcNAc and UDP-MurNAc are then linked by a 1,4 glycosidic bond. Subsequently, a family of ligases, including MurC, MurD, MurE, and MurF, catalyses the addition of amino acids to form a pentapeptide chain. This chain consists of L-alanine, D-glutamic acid, meso-diaminopimelic acid (DAP), and two D-alanine residues (Figure 2, cyan).

The Mur enzymes mediate peptide linkage formation by first activating UDP-MurNAc via acylphosphate formation. This is followed by a nucleophilic attack by the amine group of an amino acid on the carboxylic acid carbon, sequentially elongating the peptide chain (Figure 2). This step is critical for the assembly of the bacterial cell wall precursor [30,31].

## 4. MA Synthesis

**Figure 3:**
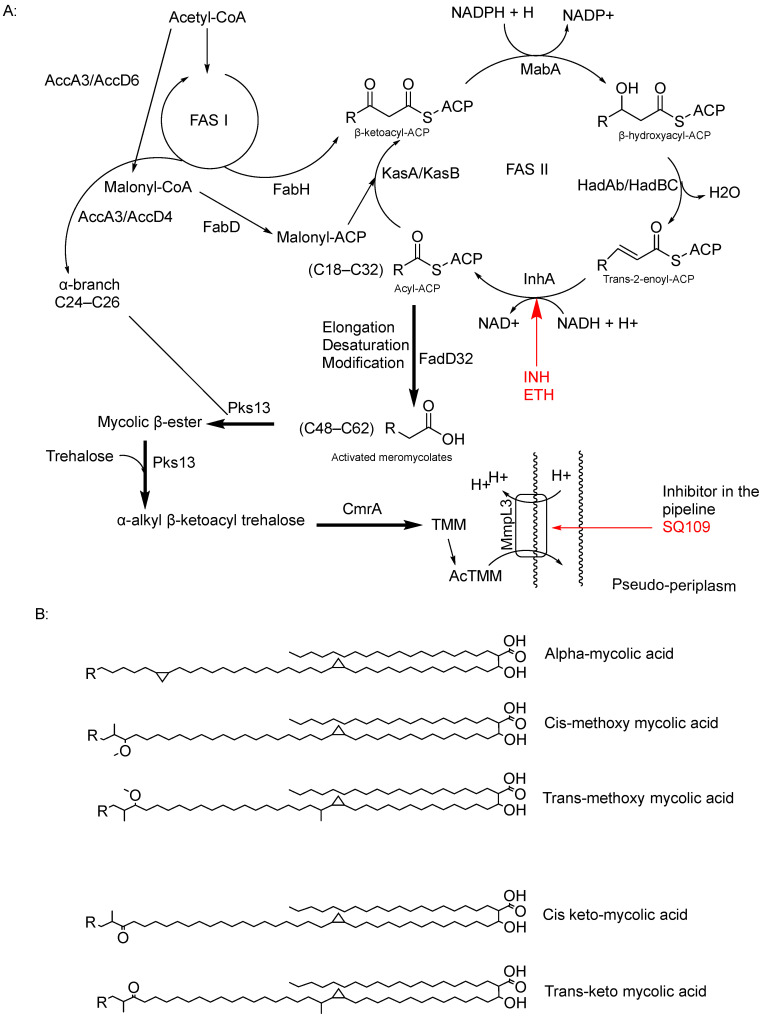
(**A**) FAS I and FASII pathways to form the mature MA, displaying the reduction function of InhA within the FASII system, including its inhibitors (INH and ETH); on the other hand, delamanid has been proved to inhibit methoxy and keto mycolates, but there is no currently identified target. And there is also the function of the MmpL3 transporter and its promising inhibitor SQ109. (**B**) Chemical structure of mycolate classes [35].

MA was first extracted from bacteria by Anderson and reported in the Journal of Biological Chemistry in 1927 [32]. However, a comprehensive understanding of its overall structure only emerged in the 1960s [32,33,34]. MA is a type of long-chain α-alkyl-β-hydroxy fatty acid composed of C42–C62 meromycolate units and a saturated α-chain ranging from C24 to C26. It is attached to AG and outer lipid layers (Figure 1). Mycolates are classified based on the presence of functional groups such as keto or methyl groups on the fatty acid chain and their configuration (trans/cis), as well as the length of the chain, which varies due to genetic and environmental factors in mycobacteria (Figure 3) [18,23,33].

The synthesis of MAs begins in the cytoplasm through a series of condensation reactions between acetyl-CoA and malonyl-CoA. These reactions are catalysed by enzymes such as enoyl-ACP reductase (FabI), leading to the formation of 3-keto-deoxyoctanoate [36,37]. Subsequently, 3-keto-deoxyoctanoate is reduced by 3-oxoacyl-[ACP] synthase I (FabA), which transfers hydrogen atoms from NADH to the double bonds in the chain, resulting in the production of saturated fatty acids. Additionally, malonyl-CoA molecules are added to the fatty acid chain via the type II fatty acid synthase (FASII) pathway, facilitated by enzymes like InhA, which also plays a role in the reduction of the fatty acid to form ACP [37]. InhA, with its dual role in MA synthesis, is a key target for chemotherapy, notably targeted by INH and ethionamide (Table 1) [38].

Mycolyl transferase (FabZ) catalyses the transfer of reduced C12:0-CoA to the ACP, forming the complex 3-hydroxydecanoyl-ACP. This complex undergoes further modification, catalysed by mycolyl transferase/polyketide synthase, converting it into 3-hydroxydecanoyl-AMP by forming a thioester bond with adenosine monophosphate (AMP) and the initially synthesised fatty acid chain [23,36,39,40].

Finally, MA synthase converts 3-hydroxydecanoyl into MA through a series of condensation, dehydration, and reduction reactions [39]. Delamanid, the last-approved drug, has shown effectiveness against MA synthesis by inhibiting this final step in the synthesis [12].

MAs are hydrophobic fatty acid chains that contribute to mycobacterial virulence by blocking hydrophilic drugs and enhancing survival within the host, especially inside macrophages. Their waxy lipid layer also strengthens the cell wall, increasing bacterial resilience [41]. Targeting the synthesis of MAs has proved to be an effective strategy in TB chemotherapy.

## 5. AG Synthesis

AG is a heteropolysaccharide that plays a crucial role in the structure of the mycobacterial cell envelope. It forms covalent bonds with muramic acid residues of PG through phosphodiester bonds [42]. Together, PG and AG account for approximately 35% of the cell envelope, acting as a copolymer that spans between the inner plasma membrane and the MA layer (Figure 1). The overall structure of AG in Mtb consists of a linker unit that attaches the molecule to PG, AG, and arabinan domains containing approximately 30 β-D-galactofuranosyl (Galf) residues.

### 5.1. Synthesis of the Linker Unit

**Figure 4:**
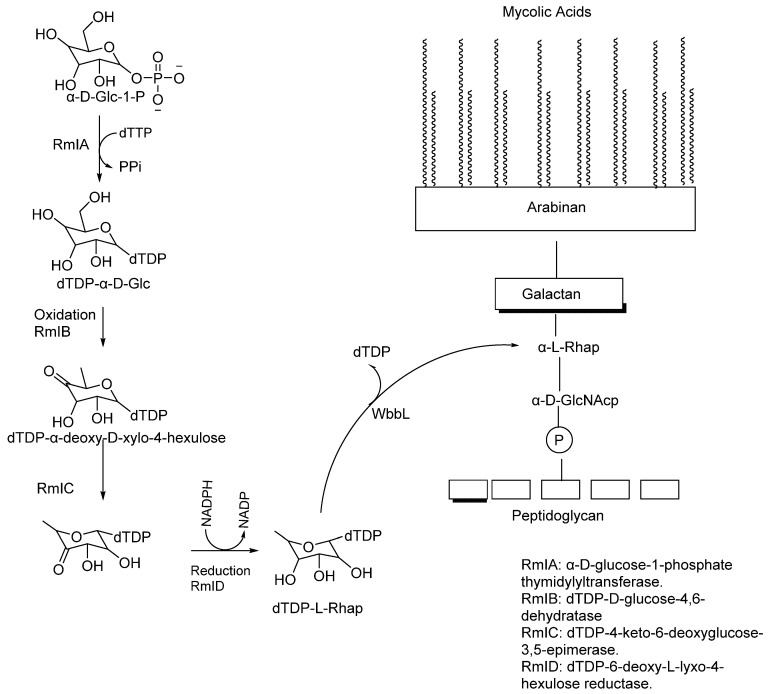
Four-step reaction of the RmI family of enzymes to make dTDP-L-Rhap, followed by the action of WbbL that cleaves dTDP to form α-L-Rhap [46].

The biosynthesis of the linker unit starts with the conversion of GlcNAc-1-P from UDP-GlcNAc to the lipid carrier (C50-P) by *N*-acetylglucosaminyl phosphate transferase (WecA) (*Rv1302*) [43,44]. The WecA enzyme shows homology in Mtb and plays a major role in Gram-negative organisms like *E. coli*, where it catalyses the first step in lipopolysaccharide O-antigen synthesis. Some other transmission electron microscopy studies have shown the important role WecA plays in *M. smegmatis* (MSMEG_4947) growth [44]. Alternatively, the rhamnosyltransferase WbbL enzyme, encoded by *Rv3265c*, has been shown to play a significant role in the AG–PG linker synthesis; Mills et al. investigated the importance of this enzyme for mycobacterial growth by temperature-sensitive *M. smegmatis* mutants (Figure 4) [45].

Finally, the rhamnose unit connected to the 5-β-D-Galf is biosynthesised by converting α-D-glucose-1-P and TTP into dTDP-rhamnose by RmlA–RmID enzymes in a four-step synthesis [47,48].

### 5.2. Galactan and Arabinan Biosynthesis

Galactan is a linear polymer, composed of about 20 to 30 galactose units [49]. It is attached to the PG polymer via the linker (Figure 4), which acts as a primer for galactan synthesis. Using decaprenyl phosphate as a lipid carrier, galactan is synthesised on the cytoplasmic side of the plasma membrane. GIf1 initiates galactan synthesis by attaching the first and second Galf to glycolipid2 via β-(1,4) and β-(1,5) glycosidic bonds. Galf2 then produces decaprenyl-p-p-GlcNAc-Rha-Galfx, a lipid-linked galactan polymer with 20–30 alternating β-(1,5) and β-(1,6)linked β-D-Galf [50]. The galactan polymer is translocated from the cytoplasm to the periplasm by the ABC transporter Wzm-Wzt [51].

Arabinan polymer synthesis is initiated by the arabinofuranosyltransferase (AftA) locus (*Rv3792*), which catalyses the addition of Araf in the sugar donor DPA into the galactan chain at positions 8, 10, and 12. DPA is derived from 5-phosphoribosyl-1-pyrophosphate, converted to decaprenylphosphoryl-5-phosphoribose by UbiA (*Rv3806c*) [52]. This addition acts as a primer to extend the arabinan chain by adding more 2-β-D-Araf [53].

Further polymerisation of the arabinan chain is carried out by the embA and embB (*Rv3794*, *Rv3795*) enzymes, which have 1.5 transferase activity. These emb enzymes are also crucial for forming the terminal hexa-arabinofuranosyl motif [54]. Zhang et al. identified that embA and embB form a heterodimer and catalyse the formation of the branched (1→3) arabinofuranosyl linkage on the 3-0 of the third arabinosyl residue of the pentamer chain; on the other hand, embC forms a homodimer which catalyses from (1→5) positions [55].

The five approved Mtb drugs will be discussed, with a focus on their structure–activity relationships (SARs) supported by 2D docking models, while also addressing concerns related to clinical trial outcomes.

## 6. Cycloserine (CS)

**Figure 5:**
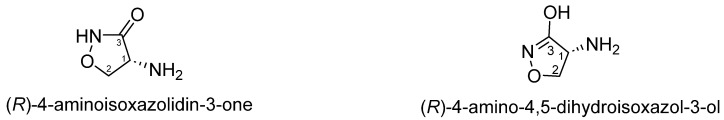
Chemical structure of D-cycloserine (R-4-aminoisoxazolidin-3-one) and its tautomer.

Schatz and Waksman discovered the presence and effectiveness of CS while studying soil-dwelling bacteria [56]. It was extracted from *Streptomyces garyphalus*, *Streptomyces lavendulan*, *Streptomyces roseochromogenus*, and *Streptomyces orchidaceous* [56,57,58]. CS is a cyclic analogue of D-alanine, featuring an isoxazolidinone ring with a primary amine attached to a chiral centre crucial for its activity (Figure 5).

CS forms a structural isomer believed to be the active form of the drug. Its efficacy against Mtb led to its expedited approval by the United States Food and Drug Administration (FDA) in 1968 for the treatment of Mtb infections [11].

**Figure 6:**
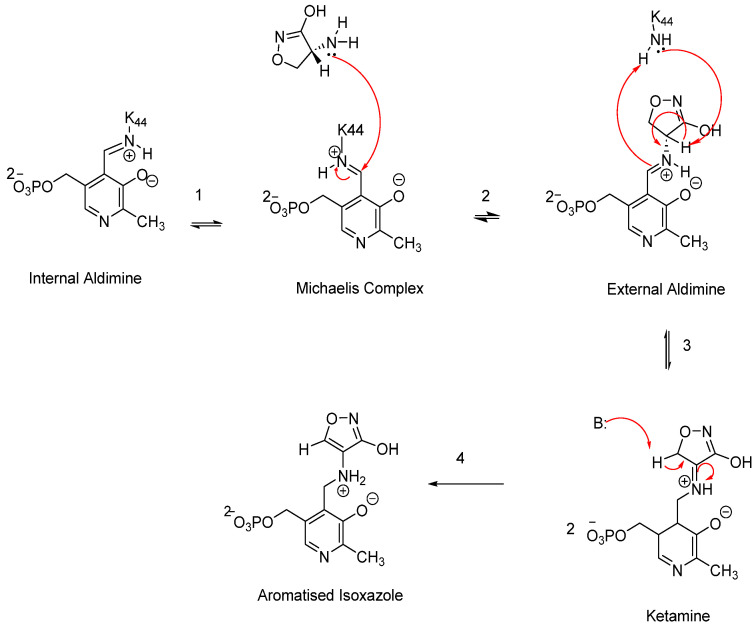
A schematic representation of the proposed CS mechanism of inhibition.

CS functions as a structural analogue of D-alanine, halting the conversion of L-alanine to D-alanine. This disruption is critical for PG formation in both Gram-positive and Gram-negative bacterial species, thereby contributing to CS’s broad-spectrum activity [59]. CS inhibits PG synthesis by irreversibly binding to the pyridoxal 5-phosphate cofactor (PLP) in the Alr enzyme, forming a stable oxime (Figure 6). This mechanism of action disrupts bacterial cell wall synthesis.

The proposed mechanism of action for CS was elucidated in 1998 based on studies of its deactivation of the PLP cofactor in several enzymes, including Alr, γ-aminobutyric acid aminotransferase, and D-amino acid transferase, which was termed the “aromatisation” mechanism [60,61]. In the normal physiology of Alr, the PLP cofactor is bound within the enzyme’s active site via a lysine side chain through an aldimine linkage. CS disrupts this process by binding to the alanine side chain, displacing the lysine. This interaction begins with the tautomer of CS ((R)-amino-4,5-dyhydroisoxazol-3-ol) initiating a nucleophilic attack on the aldimine linkage of the PIP cofactor, forming an external aldimine (Figure 6). In the subsequent step, stabilisation of the intermediate occurs, followed by another base reaction with ketamine, resulting in the formation of a highly stable aromatised isoxazole complex. This final complex is irreversible in vivo, effectively preventing the conversion of L-alanine to D-alanine and thereby blocking PG synthesis [59,62].

On the other hand, Prosser et al. suggest that CS more strongly inhibits Ddl [63]. This study evaluated metabolites present in the media after CS administration. However, ambiguity persists in the literature regarding whether Alr or Ddl is the primary lethal target of Mtb, as binding affinity comparisons do not definitively determine the primary lethal target, along with the absence of enzyme crystal structure studies. Nevertheless, pathogenesis studies of Mtb underscore the essential roles of both enzymes in PG synthesis [59].

Recently, there has been interest in using CS for the treatment of depression and other brain disorders due to its partial agonism on the N-methyl-D-aspartate (NMDA) receptor, which has shown promising outcomes in individuals with major depressive disorder [64,65]. However, CS’s broad-spectrum activity suggests it may disturb gastrointestinal flora regulation or foster resistant strains, weakening this proposal.

Structural modifications are feasible for CS, as it meets Lipinski’s rule of five, with potential adjustments in size, hydrogen bond donating/accepting sites, and logP value (partition coefficient between oil and water). These standard criteria are essential in drug design and optimisation [66,67].

### CS Toxicity Concerns

In 2019, the use of CS was designated for cases where first-line therapy proves ineffective, a decision supported by contemporary clinical studies. One such study, a nationwide retrospective cohort study conducted in China in 2019, reported positive outcomes from clinical trials [68]. The study evaluated the efficacy and safety of CS, underscoring its importance in the treatment of MDR-TB as part of a multi-drug regimen. The results were positive, showing that treatment regimens containing CS led to statistically significant improvements in MDR-TB treatment outcomes, with a cure rate of 66.0% [68]. However, psychotic symptoms affected 4.3% of patients treated with CS-containing regimens [68].

Initially, psychiatric symptoms associated with CS were considered negligible as they typically resolved upon discontinuation of the drug. However, in 1965, an article published in the Canadian Medical Association Journal reported the first case of chronic psychotic symptoms linked to CS use [69]. The underlying mechanism through which CS caused these symptoms remained unclear at the time [69]. In 1991, Emmitt et al. measured the levels of cyclic guanosine monophosphate (cGMP) following the in vivo administration of CS. They observed elevated levels of cGMP, which suggested that CS acts as a partial agonist on the NMDA receptor. Overactivation of this receptor disrupts the normal regulation of neuronal impulses, potentially explaining the psychotic symptoms observed in 4.3% of patients [69,70,71,72].

On the other hand, the Chinese cohort study has some limitations, such as its focus on a specific population group without considering other ethnicities. This omission could overlook new polymorphisms in genes like Alr (Gly122Ser) or the alanine transporter gene CycA (*Rv1704c*), which may lead to different pharmacokinetic profiles [73,74]. Furthermore, the retrospective nature of the study means it relies on existing data, potentially impacting the randomisation and selection process of candidates [75].

In conclusion, conducting new clinical trials may not be necessary given our current understanding of CS’s pharmacodynamic effects and its associated psychotic side effects. However, developing a new generation of CS that avoids binding to the glycine site of the NMDA receptor represents a significant advancement. This could potentially elevate CS to a first-line therapy option, improve patient compliance through easier monitoring, and enhance profitability [72].

## 7. Isoniazid (INH)

**Figure 7:**
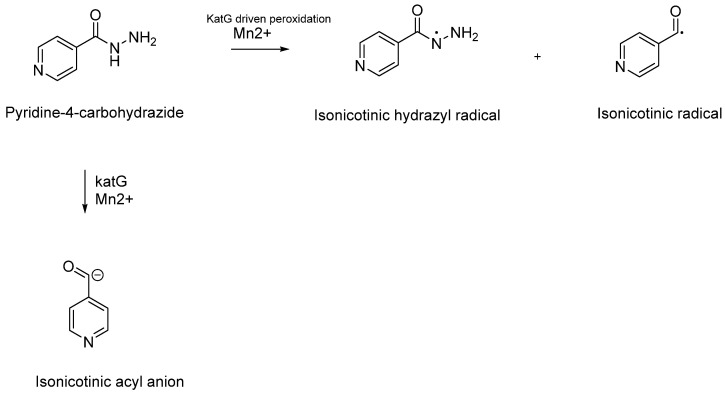
Schematic representation of the catalytic reaction facilitated by the katG enzyme involving the peroxidation of isoniazid (INH), leading to the formation of electron-rich and highly reactive species such as isonicotinic hydrazyl and isonicotinoyl radicals. An alternative pathway proposed in 1985 by Shoeb et al. suggests the formation of an isonicotinic anion [78,79,80].

Isoniazid, also known as INH or isonicotinylhydrazide, is a synthetic hydrazide derivative and a pivotal prodrug designed to combat Mtb infections [76]. First synthesised by Feldman in 1951, it was approved as the first-line treatment for Mtb infections by the FDA in 1953 [77]. INH is characterised by its ability to passively diffuse through the complex barriers of mycobacteria, ultimately entering the bacterial cytoplasm (Figure 1). Once inside the mycobacterial cytoplasm, INH requires activation by the enzyme catalase-peroxidase (katG) (Figure 7).

**Figure 8:**
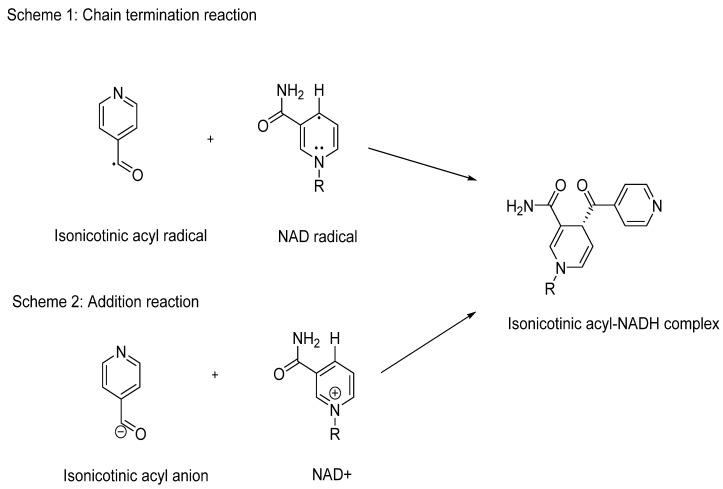
Schematic representation of the behaviour of the isonicotinic acyl radical and anion to form the inhibitory isonicotinic acyl-NADH species.

The electron-rich radicals formed during the peroxidation process of INH are believed to be its active form. Initially identified through electron spin resonance, these radicals were later confirmed by Sacchettini and colleagues, who discovered the presence of the isonicotinic acyl–NADH adduct within the active site of InhA using crystal structure studies. This in vitro research demonstrated a covalent interaction between the active form of INH and the nicotinamide head of nicotinamide adenine dinucleotide (NAD+) (Figure 8) [81,82].

The formation of the isonicotinic acyl–NADH adduct occurs through Minisci’s addition, involving the radical or anion of INH and NAD+. This process results in the creation of a potent inhibitory species that effectively blocks the normal function of InhA. Inhibition of InhA disrupts the elongation of the MA chain, crucial for the synthesis of the mycobacterial cell wall (Figure 8) [81,82]. This mechanism underscores the effectiveness of INH in combating Mtb infections by targeting essential cellular processes.

**Figure 9:**
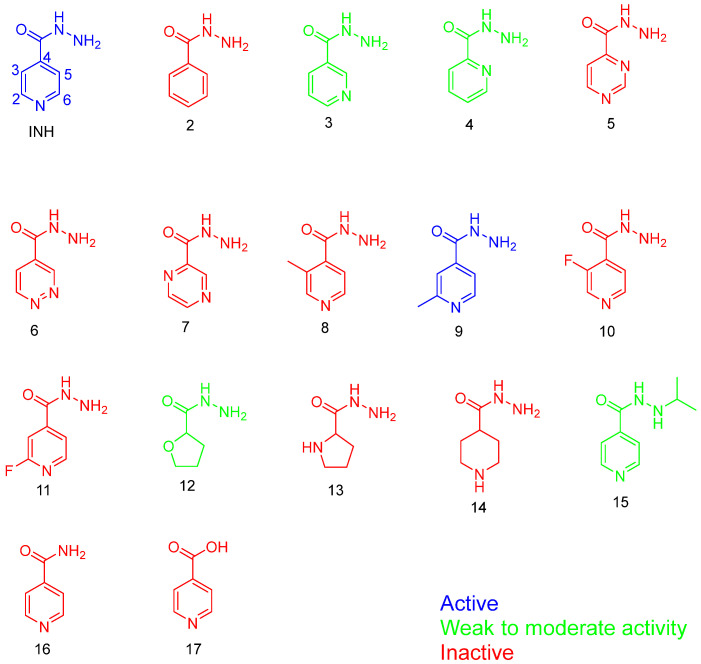
The analogues used in the Pooja et al. study [84].

When considering the SARs of INH, the hydrazide group (CONHNH_2_) is crucial for its activity against Mtb. Therefore, any optimisation of INH must carefully preserve this hydrazide moiety. Pooja et al. explored the SARs of INH using sixteen analogues [83], assessing their minimal inhibitory concentration (MIC) against five different Mtb lineages. One critical aspect evaluated was the importance of the tertiary nitrogen in the pyridine ring of INH. To investigate this, they synthesised an analogue (# 2, Figure 9) where the pyridine ring was replaced by benzene. As expected, this alteration resulted in loss of activity compared to the standard INH, which typically exhibits MIC values ranging from 0.03 to 0.1 µg/mL across Mtb strains. This study highlights the indispensability of the pyridine ring and the hydrazide group for the antimycobacterial activity of INH.

In their study, Pooja et al. explored various analogues of INH to understand the SARs crucial for its antimycobacterial activity [83]. They found that altering the position of the nitrogen atom (analogues 3 and 4, Figure 9) resulted in weak or ineffective activity against Mtb. This underscores the specific importance of the hydrazide group’s position in maintaining INH’s potency.

Furthermore, replacing the hydrazide group with a carboxylic acid or amide (analogues 16 and 17, Figure 9) completely abolished microbial activity, emphasising the essential role of the hydrazide moiety in INH’s mechanism of action. Interestingly, analogue 9, which featured a methyl group at the 2-position of the aromatic ring, exhibited comparable potency to INH. This compound also demonstrated moderate activity against other mycobacterial strains, such as *Mycobacterium avium* (12.5 µM). However, its weaker activity against XDR-TB suggests the need for further investigation into its interaction with resistant targets and the characterisation of resistant strain structures.

Increasing the side chain length is a conventional strategy in drug design to enhance lipophilicity (logP), potentially improving binding affinity. Analogue 9 of INH could be optimised further by extending the side chain at position 2. This modification is expected to preserve the catalytic site (hydrazide group) and maintain the formation of radicals crucial for InhA binding. Exploring this approach may enhance the efficacy of INH analogues against drug-resistant TB strains [85].

Unlike CS, which raises concerns about off-target toxicity, INH is generally safer in terms of side effects. However, resistance remains a significant challenge with this drug [85,86]. While INH is highly effective as a bactericidal agent, genomic studies have identified mechanisms of resistance in Mtb, notably mutations in the katG gene [86,87]. Mutations such as the substitution of serine at position 315 by threonine are a primary cause of resistance to INH. Additionally, mutations affecting the target enzyme InhA can lead to resistance by altering its expression levels [86,87].

Over the years, computational approaches and docking studies have been instrumental in identifying INH’s primary targets and understanding its interactions with other potential targets. For instance, Lingaraja et al. conducted a study comparing wild-type and mutant types (S315T and S315N) of katG enzymes [88]. They observed abnormal hydrogen bonding between the secondary amine of INH and Thr315/Asn315 in mutant katG enzymes, which contrasts with the wild-type control. Despite similar binding energies between wild-type and mutant katG (wild = −5.36, mutant = −4.98 kcal/mol), Mathiesen et al. demonstrated that this hydrogen bond may scavenge radicals, inhibiting the conversion of INH into free radicals (Figure 8) [89]. Moreover, docking studies have highlighted that the INH–NAD adduct is a more potent inhibitor of InhA than INH alone. This computational finding aligns with the in vitro studies conducted by Nguyen and co-workers [90].

While katG mutations are the primary cause of resistance to INH, further docking studies are needed to elucidate how the INH adduct interacts with various InhA mutants. This will provide insights into the structural changes in the mutant InhA pocket and aid in designing new drugs based on these interactions [91].

## 8. Ethionamide

Thionamides, specifically ethionamide (pyridine-4-carbothioamide) and prothionamide (2-propylpyridine-4-carbothioamide), are second-line agents currently used to combat MDR-TB. Recently, they have gained popularity due to the increased prevalence of MDR-TB [92].

**Figure 10:**
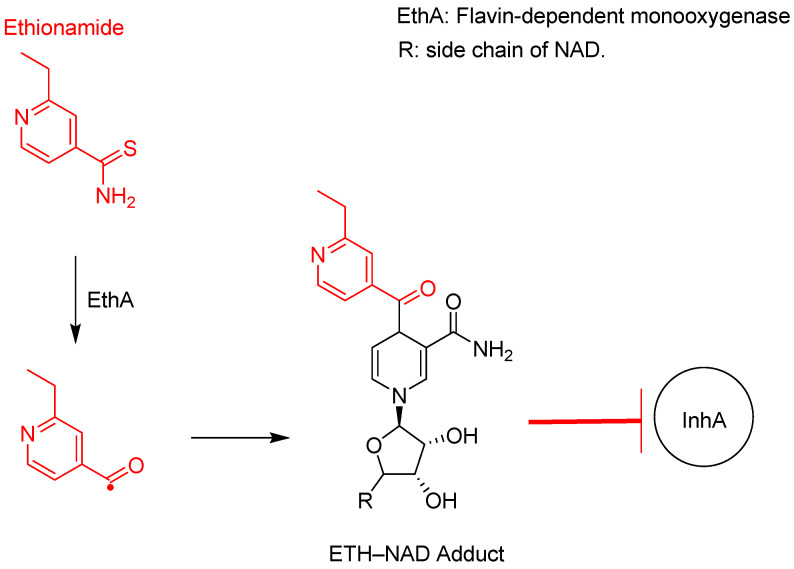
Ethionamide activation to an active radical species by EthA, which complexes with NAD to form a strong inhibitory adduct to InhA [91,93].

Thioamides are prodrug analogues of INH that also target MA synthesis by inhibiting InhA, but through a different metabolic pathway (Figure 10). Ethionamide, an analogue of INH, was developed in the late 1950s and received FDA approval in 1968 [10].

As shown in Figure 7 and Figure 10, both ethionamide and INH require activation to a highly reactive radical species; however, different enzymes catalyse this reaction. EthA catalyses the activation of ethionamide through a deamination reaction, whereas INH loses its hydrazine group.

**Figure 11:**
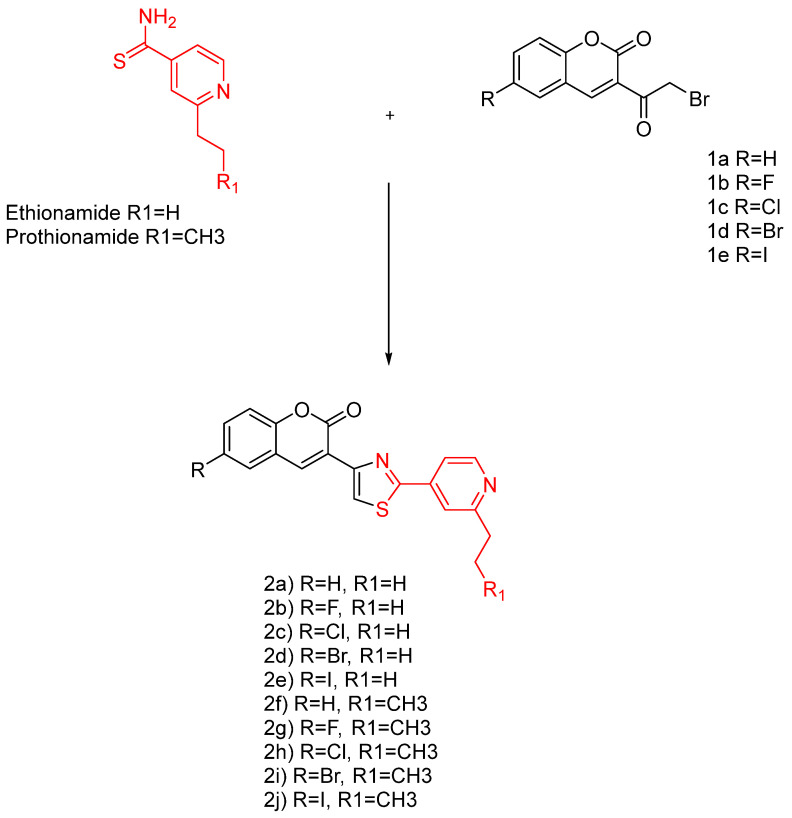
The ethionamide and prothionamide analogues [94].

Like INH, ethionamide has a potential structural modification at the 2-position carbon in the pyridine ring, and the extension of an extra carbon chain was successful in developing prothionamide (PTH). Recently, ETH- and PTH-based coumarinylthiazole derivatives merged in drug design (Figure 11).

Compounds 4a–4j displayed equal/or better anti-TB potency than INH and PTH. Specifically, compounds 4a, 4b, 4f, and 4g are equipotent to INH and PTH, whereas 4c–4e and 4h–4j displayed better MIC values than INH and PTH. This discovery indicates the possibility of the coumarin–thiazole–pyridine nucleus as a potential pharmacophore.

The mentioned analogues are shown to have a better selectivity profile than the pharmacophore (Figure 11, red) and exhibit a strong inhibition to DprE1, thus blocking the MA synthesis. Similar compounds were reported in the literature, lacking the pyridine ring, with MIC values ranging from 15 to > 663 g/mL and 6.25–25 g/mL against Mtb *H37Rv* [95,96].

### Ethionamide and INH Clinical Considerations

Ethionamide exhibits an MIC against Mtb ranging from 0.6 to 2.5 mg/mL, demonstrating its effectiveness as an anti-TB agent [97,98]. One of the main advantages of ethionamide is its approximately 100% oral bioavailability. After oral administration, nearly all of the drug reaches systemic circulation without being significantly altered or degraded by metabolic processes in the liver [99].

Unlike INH, which is known to inhibit various cytochrome P450 (CYP450) isoforms—specifically CYP1A2, CYP2A6, CYP2C19, and CYP3A4—ethionamide does not undergo first-pass metabolism [100]. This distinction is clinically important as it reduces the risk of drug–drug interactions that can complicate treatment regimens. The metabolic profile of ethionamide makes it a safer alternative compared to INH in terms of potential interactions with other medications. However, despite these benefits, ethionamide has not received approval from the Medicines and Healthcare Products Regulatory Agency (MHRA) and thus remains a second-line therapy according to the FDA. This lack of approval is largely attributed to numerous reports linking ethionamide to severe cases of hypothyroidism, raising concerns about its safety profile in clinical use [101,102].

On the other hand, besides hepatoxicity via the metabolism of INH, INH-induced peripheral neuropathy is also common. This is mainly due to the metabolite of INH being a strong inhibitor of pyridoxine phosphokinase, which catalyses the activation of pyridoxine to pyridoxal-5-phosphate. Another observed mechanism is due to the promotion of pyridoxine excretion at high doses of INH [103]. Therefore, co-prescribing pyridoxine with INH has been shown to be effective in preventing/treating INH toxicity [103,104].

## 9. Delamanid

**Figure 12:**
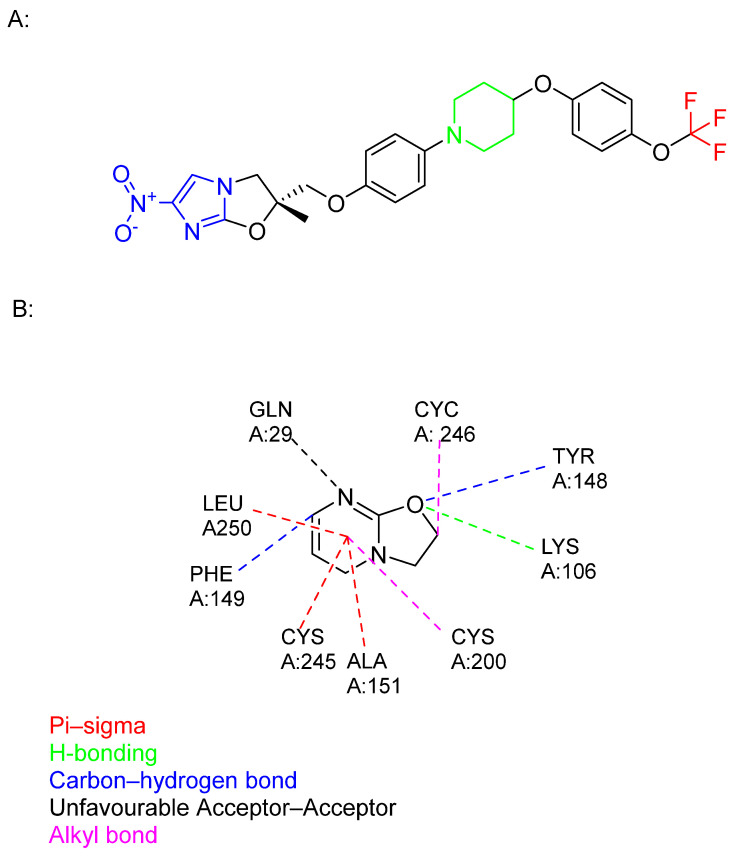
(**A**) The chemical structure of delamanid: nitroimidazole core (blue), trifluromethoxy group (red), hydrophobic part (black), and piperidine (green). (**B**) The structure of an identified delamanid metabolite, displaying various interactions of this ligand with human serum albumin [107].

Delamanid (Deltyba, OPC67683) belongs to the bicyclic nitro-dihydro-imidazooxazole class of compounds, specifically designed to inhibit the synthesis of keto and methoxy MAs without affecting α-mycolate structures (Figure 12). It was first discovered by the Japanese company Otsuka Pharmaceutical Co., Ltd. through random screening efforts in 2013, following the initiation of their TB discovery programme in the early 1990s [105]. Delamanid received approval from the Japanese Medicinal Devices Agency in 2014 for the treatment of adult pulmonary MDR-TB, and the European Medicines Agency (EMA) granted approval in the same year [12,106].

The SARs of delamanid revolve around its core moieties: nitroimidazole and trifluoromethoxy groups, which are crucial for its antimycobacterial effects. Moreover, hydrophobic aromatic rings bound to the piperidine linker further enhance both efficacy and safety [18,108].

Despite its selectivity in targeting methoxy and keto mycolates, indicating a different mechanism from that of INH, the exact target remains unknown. Recently, a metabolite, identified as a product of serum albumin metabolism, was found to have a higher affinity for albumin, suggesting it may have greater therapeutic potential than the parent compound (Figure 12) [107,109].

**Figure 13:**
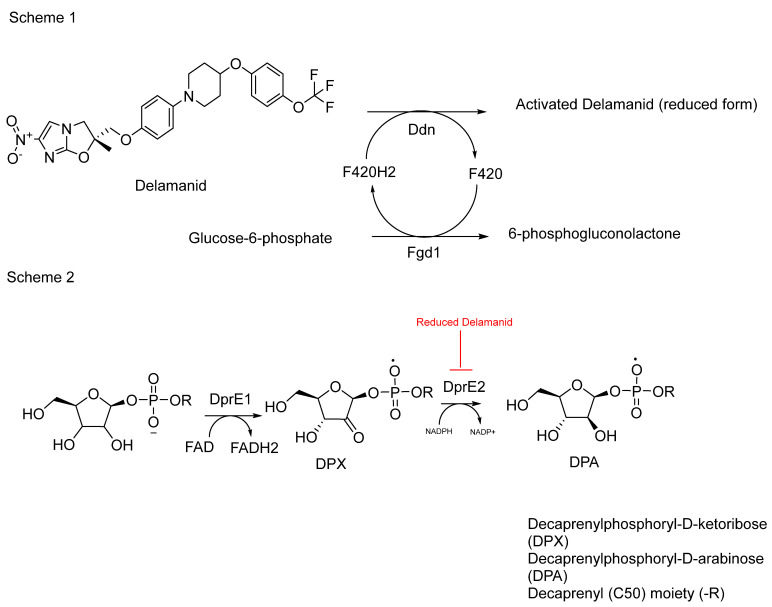
Schematic representation 1 shows the activation of delamanid by the F420H2 cofactor, which is produced by ferredoxin-NADP+ oxidoreductase 1 (Fgd1) through the conversion of glucose-6-phosphate to 6-phosphogluconolactone. Ddn uses the protonated F420H2 to activate delamanid, releasing F420. Scheme 2 shows the conversion of DPX to DPA via the DprE2 catalysed reaction which is shown to be a target for delamanid [113].

Delamanid functions as a bioprecursor prodrug, a classification first proposed by Wermuth, due to its unintentional synthesis as a prodrug lacking a transport moiety [110]. According to Singh and colleagues, delamanid is bioactivated by nitroreductase, specifically deazaflavin (F420)-dependent nitroreductase (Ddn) (*Rv3547*) [111,112]. Recent findings have shed light on the mechanism of action of delamanid [113]. The researchers discovered that the activated form of delamanid inhibits the function of the enzyme DprE2, crucial for the formation of decaprenylphosphoryl-D-arabinose (DPA) (Figure 13). This discovery represents a significant step forward in understanding how delamanid disrupts mycobacterial cell wall synthesis, contributing to its efficacy against multidrug-resistant tuberculosis.

The effectiveness of delamanid has been demonstrated through a study comparing its impact on *Mycobacterium bovis* BCG strains with overexpressed DprE2 enzyme against a control group with an empty vector. The results indicated a substantial reduction in activity, up to 20-fold lower, compared to the control group [113]. This underscores delamanid’s ability to inhibit DprE2, which plays a crucial role in the synthesis of decaprenylphosphoryl-D-arabinose (DPA) (Figure 13).

While delamanid is known for its inhibition of methoxy and keto mycolates, suggesting a specific target, recent studies indicate broader actions. DPA serves as a major precursor in the synthesis of AG, a critical component of the mycobacterial cell wall [114,115]. Inhibiting DPA synthesis could potentially destabilise the cell wall by reducing AG levels. This disruption weakens interactions between MAs and AG, diminishing the integrity of the cell wall structure [18].

Despite strong evidence supporting its mechanism, identifying the structure of delamanid’s active metabolite and its target remains a priority. Both sides of the drug, the trifluoromethoxy group and nitroimidazole, are crucial for its antimicrobial activity (Figure 13). It is speculated that delamanid may produce two active metabolites upon cleavage by Ddn, each potentially targeting different aspects of MA synthesis and DprE2. This hypothesis stems from the drug’s dual functionality and the complex enzymatic pathways involved in MA biosynthesis.

However, concerns about potential resistance with long-term delamanid use persist. Genetic evidence suggests the emergence of nonsynonymous mutations affecting the Ddn enzyme, which is essential for delamanid activation [116]. Understanding these resistance mechanisms is crucial for managing treatment efficacy and developing strategies to mitigate resistance in TB therapy.

### Key Clinical Considerations in Delamanid Use

Delamanid is a potent antimycobacterial agent that does not affect Gram-positive/negative bacteria; this is advantageous in preventing wider use and thereby reducing resistance. Animal studies have reported a bioavailability of 35–60% for delamanid, with the drug primarily protein-bound and metabolised via plasma albumin. To a lesser extent, it is metabolised by CYP3A4, CYP1A1, CYP2D6, and CYP2E1. However, numerous in vitro hepatocyte studies have shown that delamanid and its metabolites are not substrates of CYP enzymes [117,118].

In animal studies, delamanid was found to be safe, with no reported central nervous system (CNS) or respiratory toxic effects at a dose of 100 mg twice daily. However, in vitro studies indicated that delamanid inhibits cardiac potassium channels. Evidence shows that delamanid metabolites block HEK-293 and CHO-K1 cells expressing these voltage-gated potassium channels. In addition to causing electrolyte depletion, delamanid has been shown to induce QT prolongation, which is exacerbated by vomiting, a common side effect of the drug [119,120].

Another concern in delamanid use is the blocking of vitamin K1 production. Multiple data have reported the resulting reduction of clotting factors (II, VII, IX, and X) and increased prothrombin time and partial thromboplastin time [121]. Although the older generation of bicyclic nitroimidazoles had teratogenic effects, which led to their withdrawal as potential TB agents, delamanid was the optimised lead that was shown to eliminate mutagenicity [122]. Despite this, delamanid was found to be teratogenic in in vivo studies on rats, although the concentration used was much higher than what is clinically administered. Additionally, concerning data have revealed that delamanid is present in breast milk at a concentration four times higher than in the blood [123].

## 10. Ethambutol

**Figure 14:**
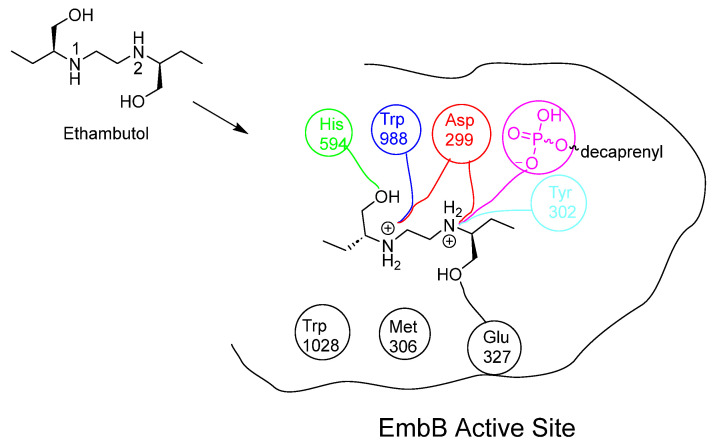
Chemical structure of EMB and the interactions that EMB performs within the embB active site [55]. EMB’s two hydroxyl groups form H-bonds with His594 and Glu327: both secondary amines are ionised at the acidic mycobacterial pH. Imino 1 forms a strong electrostatic interaction with Asp299 and Trp988. Alternatively, imino 2 forms electrostatic interactions with Asp299, Tyr302 and the deionised hydroxyl group on the phosphate carrier [55,128].

EMB, also known by its brand names Myambutol, Servamutuol, and Etibi, is a member of the ethionamide family of drugs (Figure 14). It was first synthesised by Siemens and colleagues in 1961 at Lederle Laboratories [124]. The effectiveness of EMB against Mtb was initially demonstrated in various studies that showed positive outcomes in Mtb-infected mice and guinea pigs [125,126]. These successful preclinical studies paved the way for subsequent clinical trials that ultimately led to the approval of EMB by the FDA in 1968 [127].

EMB acts as a bacteriostatic agent by inhibiting arabinosyl transferase enzymes, which are responsible for polymerising arabinose into arabinan, an essential component of AG synthesis [55]. By blocking this process, EMB disrupts the synthesis of AG, which is essential for the integrity and permeability of the mycobacterial cell wall.

In summary, EMB plays a pivotal role in TB treatment by targeting the synthesis of AG, thereby impairing the structural integrity of Mtb’s cell wall and inhibiting its growth and propagation.

EMB exhibits multifaceted actions due to its inhibition of both embB and embC enzymes, which play distinct roles in the synthesis of the mycobacterial cell wall. These enzymes are integral membrane proteins in mycobacteria, crucial for the formation of α (1→3) bonds in the hexa-arabinofuranosyl motif of AG. Specifically, embA and embB enzymes contribute to this linkage, while embC is involved in forming α (1→5) glycosidic bonds that elongate the LAM chain. LAM is important for mycobacterial survival, as it interacts with host mannose receptors, thereby slowing down phagosomal maturation [129,130].

Initially, embA and embB were identified as targets of EMB in *Mycobacterium avium* (*M. avium*) in 1996 [14]. Subsequent studies extended this finding to include emb proteins in Mtb [131]. However, recent advancements, such as cryo-electron microscopy studies have challenged earlier beliefs, suggesting a more complex mechanism of action for EMB [55]. Zhang’s research indicated that EMB does not bind to embA-encoded arabinosyl transferase, suggesting a different mode of action than previously understood [55]. Genomic studies have further supported Zhang’s findings, revealing that mutations leading to structural alterations in embB and overexpression of embC are primarily responsible for EMB resistance in Mtb [14,131,132,133,134].

This resistance mechanism underscores the importance of understanding the specific targets and structural alterations that contribute to drug resistance, guiding efforts in TB treatment strategies.

### Clinical Concerns in Ethambutol Administration

Early clinical trials involving ETB (a compound under investigation) were found to be inaccurate, primarily focusing on its acute effects. A study examined both the initial treatment and retreatment with ETB over a four-year period, involving a total of 145 patients [135]. Although the S-isomer of ETB demonstrated effectiveness, the trial utilised the racemic mixture for its investigations [125]. The study reported no acute liver toxicity associated with ETB, even among alcoholic patients diagnosed with portal cirrhosis. However, there was an instance of ocular toxicity in one patient, which was suggested to be related to dosage levels.

Subsequent research indicated that the incidence of dosage-related toxicity varied significantly; 18% of participants experienced ocular neuritis at a dosage of 35 mg/kg/day, while only 1% experienced this side effect at a lower dosage of 15 mg/kg/day [136]. Currently, the R-form of ETB is recognised as toxic and is not included in the formulation used for treatment [137]. Advances in dosing strategies have contributed to a reduction in toxicity risks, with recommended dosages ranging from 100–400 mg for adults and 15–20 mg/kg/day for children [138].

The mechanism through which ETB induces visual toxicity has been explored in retinal ganglion cells [139]. Research indicates an excitatory response via the glutaminergic pathway, suggesting that NMDA/AMPA receptor blockers may serve as a potential adjunctive therapy to mitigate these effects. In vivo studies further support the efficacy of memantine, an NMDA receptor blocker, in preventing ETB-induced retinal damage [140].

Another proposed mechanism involves the generation of reactive oxygen species, leading to retinal cell injury and death [141,142]. In addition, the understanding of ETM toxicity mechanisms—contributing to optic neuropathy, neuritis, retrobulbar neuritis, and peripheral neuropathy—has evolved alongside insights into mitochondrial dysfunction associated with optic neuropathies [142].

Overall, most ocular symptoms can be prevented through standard dosing practices and pre-treatment ocular testing. While NMDA receptor antagonists may offer therapeutic potential, their off-label use should be restricted to high-risk patients, such as those with existing conditions like diabetes. This should follow a comprehensive evaluation of the benefits and risks to minimise the potential for polypharmacy.

## 11. The Future of Cell Wall Targeting Agents in TB Treatment

### 11.1. DprE1 Inhibitors

**Figure 15:**
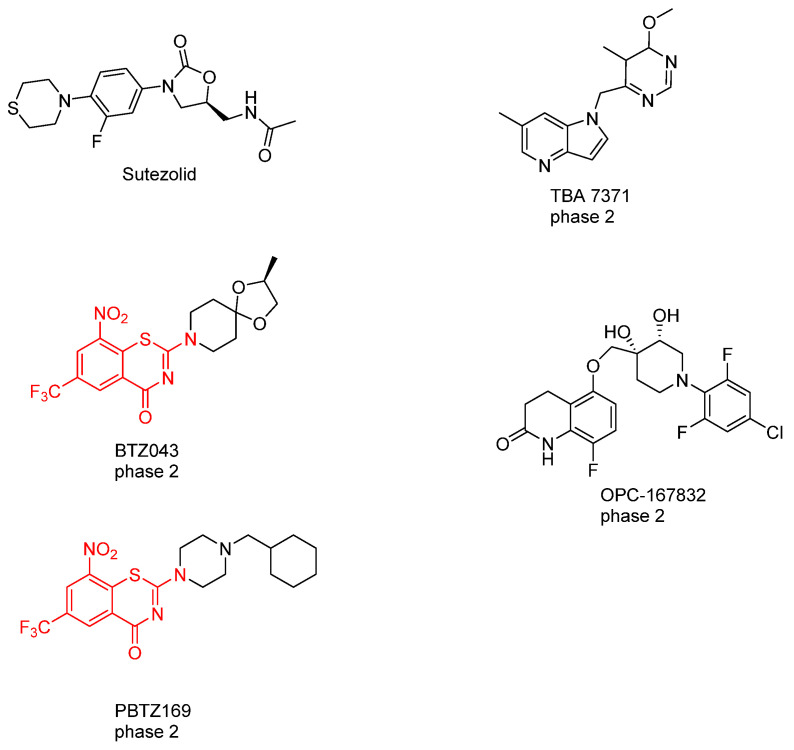
Chemical structures of the drugs in the pipeline that target DprE1. Sutezolid, BTZ043, PBTZ169, TBA-7371, and OPC-167832 [145,146].

So far, six agents have advanced to phase II clinical trials for treating MDR-TB and XDR-TB. Sutezolid, a novel oxazolidinone that inhibits protein synthesis, has been demonstrated to block the DprE1 enzyme, thereby inhibiting arabinan synthesis [143]. In addition, TBA 7371, OPC-167832 and both benzothiazinones derivatives BTZ043 and PBTZ169 have shown effectiveness in inhibiting arabinan synthesis by targeting DprE1 (Figure 15) [143,144].

Sutezolid is an oxazolidinone class drug that acts on the 23S rRNA of the 50S ribosomal subunit. It does not directly inhibit DprE1 but prevents protein synthesis by binding to this bacterial ribosomal subunit [143]. Sutezolid in vitro studies displayed an MIC ranging from 0.0625 to 0.5 mg/L. Interestingly, the metabolite of sutezolid (U-101603) displayed better activity against nonreplicating persisters than the parent compound [147]. On the other hand, the drug showed effectiveness in vivo when added to RIF, INH, and pyrazinamide, which effectively lowered the relapse rate (5% vs. 35%) [148,149].

A pharmacokinetic and pharmacodynamic evaluation involving 50 Mtb-infected patients was conducted in a phase II trial of sutezolid, using whole blood bactericidal activity as the primary outcome measure [150]. The analysis revealed that the most effective bactericidal activity, based on the modelling of plasma concentrations in patients (AUC, C-max), was achieved with a divided dosing regimen of 600 mg taken twice daily, as opposed to a single daily dose of 1200 mg. Another study demonstrated the bactericidal activity of the drug in sputum and blood [143].

TBA-7371 is an azaindole-based compound that exhibits a potency against multiple strains of Mtb ranging from (MIC= 0.76 to 3.12 µM) in rodent models of Mtb; it recently entered phase II clinical trials and is sponsored by the Bill and Melinda Gates Medical Research Institution [151]. The presence of the methyl and heteroaryl groups reduced toxicity on phosphodiesterase 6 (PDE6) and enhanced the compound’s activity [152]. TBA-7371 induces toxicity issues due to hepatic metabolism via CYP1A2, CYP2C9, CYP2C19, and CYP3A4) at doses exceeding 33 µM [153].

OPC-167832 is a 3,4-dihydrocarbostyril derivative, a non-covalent inhibitor of DprE1 which Otsuka Pharmaceuticals sponsored; it has a reported MIC value ranging from 0.24 ng/mL to 2 ng/mL against different strains of mycobacteria. It showed bactericidal activity better than BTZ-043, with mean IC50 = 0.258 µM compared to BTZ-043 (IC50 = 0.403), and PBTZ-169 (IC50 = 0.267) [145,151].

BTZ043 belongs to the benzothiazinone class of drugs, characterised by key constituents including sulphur and oxygen atoms in the thiazine ring, with a NO_2_ group, a strong electron acceptor, in position 6, and protons in positions 5 and 7 [154,155]. BTZ043 is a non-covalent inhibitor to the DprE1 enzyme, preventing arabinan synthesis by covalently binding to the Cys387 residue on the enzyme [155]. Furthermore, genetic studies have shown that mutations at Cys387 to Ser or Gly in DprE1 induce resistance to BTZ043 [154]. This cascade blocks the function of DprE1, a crucial component in the AG synthesis pathway within the cell wall of Mtb. This disruption in cell wall synthesis leads to the bactericidal properties of BTZ043 [154]. BTZ043 achieves this by acting as a suicide substrate for the reduced form of DprE1 [155]. It undergoes nitro-reduction to produce a nitroso species that specifically targets the thiol side chain of the active site Cys387 residue. This interaction forms a covalent bond, irreversibly inactivating the enzyme [154]. However, BTZ043’s efficacy in TB mouse models was lower than its in vitro potency (MIC = 1 ng/mL; 2.3 nM), likely due to the compound’s poor hydrophobic properties (logP = 2.84) [154]. This improvement led to the development of PBTZ, which features a piperazine group in its scaffold [144].

PBTZ169 is a subclass of benzothiazinones, specifically known as 2-piperazino-benzothiazones (PBTZ). It is structurally similar to BTZ043 and binds to the same Cys387 residue on DprE1, thereby activating the same pathway to inhibit arabinan synthesis [144]. The key difference between BTZ043 and PBTZ169 lies in the substitution pattern of the benzothiazinone core, which has increased complexity in PBTZ169. This modification enhances both the potency and stability of PBTZ169 compared to BTZ043.

PBTZ169 has been identified as the most favourable drug in the PBTZ class for treating TB, primarily due to its improved stability. The cyclohexyl group protects against nitroreductase attacks, and the methylene linker is positioned between two bulky groups, further contributing to its stability [144]. This SAR investigation underscores the importance of high lipophilicity in benzothiazinones, which has been shown to enhance the MIC. It emphasises the necessity of a logP value exceeding 3 for improved bactericidal activity (Table 3).

The lead compound PBTZ169, with its high logP value, may compromise the drug’s safety profile when administered in vivo due to its tendency to accumulate in adipose tissue. Additionally, such drugs often exhibit slow metabolism and clearance rates, which are predicted to increase both the half-life and potential toxicity.

### 11.2. Mycolic Glycolipid Transporter 3 as a Target

Mycobacterial membrane protein large protein 3 (MmpL3) functions by transporting MAs in the form of trehalose monomycolate (TMM), which is the precursor for dimycolate (TDM) [157,158]. MmpL3 is the only membrane protein that is essential for growth [159,160]; many genetic and depletion studies in *M. smegmatis* were found to result in the accumulation of TMM with a reduced level of TDM and mycolyl AG [157,161].

**Figure 16:**
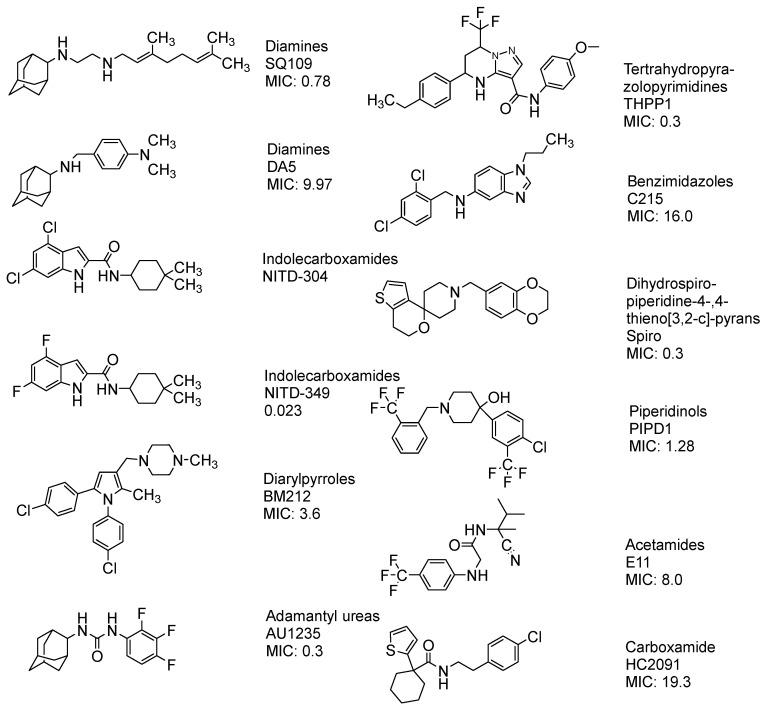
The chemical structure of the current agents showed in vitro potency to inhibit MmpL3 and its class, and MIC [157,163,164,165,166,167,168].

MmpL3 inhibitors have been identified using high-throughput whole cell-based assays which demonstrated potency in inhibiting MA synthesis [162]. Many potent MmpL3 inhibitors with diverse chemical structures have been reported in the literature (Figure 16).

Currently, the FDA has not approved MmpL3 inhibitors. As shown in Figure 16, these inhibitors lack a common pharmacophore and exhibit different scaffolding effects [169]. In the literature, many drugs have reported MmpL3 inhibition in vitro, like 2-carboxamide, pyrrole, pyrazol, benzimidazole, benzothiazolamide, and aminamide urea; however, SQ109 (ETB analogue) is the most clinically advanced in trials, completing phase IIb [170]. The treatment with SQ109 was successful when combined with first-line agents (INH and RIF), reducing the treatment time by 25–30% [94]. Despite the positive results against MDR-TB, SQ109 has a short half-life due to host drug metabolism [171].

**Figure 17:**
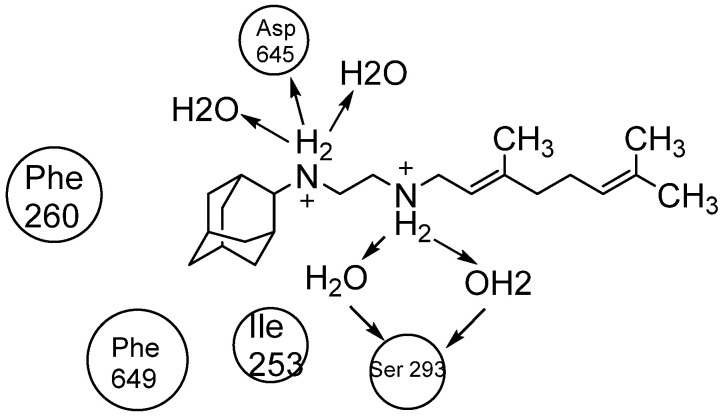
The 2D interaction of SQ109 with amino acids and water molecules in the transporter.

Interestingly, SQ109 has the same pharmacophore as ETB but shows a completely different mechanism in inhibiting cell wall synthesis. The crystal structure and binding of SQ109 in the MmpL3 transporter have been reported (Figure 17) [172].

The Desmond Simulation Interaction Diagram (SID) tool was utilised to analyse ligand interactions, revealing that SQ109 operates by allosterically binding to the water channel of MmpL3. This mechanism locks the transmembrane domain in an open conformation while keeping the pore domain closed, thereby inhibiting TMM translocation (Figure 3). This study demonstrates that SQ109 disrupts the hydrogen bonding network of water molecules, interfering with the interactions between Asp645 and Tyr257 and Asp256 and Tyr646, thereby preventing the channel from closing [172].

## 12. Conclusions

The five antitubercular agents discussed in this review are small molecules, each with potential for modification, with the aim to enhance efficacy and safety. A new generation of CS is needed to minimise NMDA receptor affinity, thereby improving safety and positioning it as a preferred first-line treatment option. Similarly, developing new analogues of INH to target mutant strains is challenging due to mutations in katG and InhA, but feasible by substitution away from the hydrazide group. Delamanid currently shows minimal resistance, yet understanding the structure of its active metabolite is crucial to identifying its primary target, possibly DprE2 or another enzyme in the FASI/FASII systems. The mechanism of action of EMB remains partially understood, with debate over whether embB or embC is the primary lethal target. Continuous genetic studies are essential to anticipate TB mutations affecting embB- or embC-coded arabinosyl transferases.

The pipeline consists of many DprE1 inhibitors with high clinical potential. In contrast, the MmpL3 agents are less clinically effective, with only SQ109 proceeding in clinical trials; despite this issue, their chemical diversity offers an interest in new effective and safe pharmacophores.

In conclusion, while screening for new anti-TB drugs is costly and less efficient, focusing on enhancing existing drugs offers advantages such as established safety profiles and long-term use experience, making this approach preferable as TB evolves.

## Figures and Tables

**Table 1:** The approved drugs that target cell wall biosynthesis.

Drug Name (Trade Name(s))	Therapeutic Target (Coded Gene)	Administration Route	Approval Year	Ref.
Isoniazid (INH, Nydrazid, IsonaRif)	Enoyl-acyl carrier protein (ACP) reductase (InhA). (*Rv1484*)	Oral, intramuscular, intravenous	1953	[9,10,11,12]
Thioamides: Ethionamide and Prothionamide (Trecator, Trecator- SC)	InhA inhibitors. (*Rv1484*)	Oral, intermuscular/IV	1968	[12,13]
Ethambutol (Myambutol, Servambutol and Etibi)	Arabinosyl transferase enzyme B and C. (*Rv3795*, *Ev3793*)	Oral	1968	[9,10,11,12,14,15]
Cycloserine (Seromycin)	D-alanine racemase (Alr) and D-alanine-D-alanine ligase (Ddl). (*Rv3423c*, *Rv2981c*)	Oral	1968	[8,9,10,11,16]
Nitroimidazole: Delamanid (Deltyba)	DprE2 enzyme. (*Rv3791*)	Oral	2014 *	[8,10,12,17]

* For adults by the Medicinal Devices Agency in Japan, followed by approval by the European Medicines Agency (EMA) in the same year.

**Table 2:** The CDC and NICCE guidelines for the diagnosis and treatment of active and latent TB.

Clinical Considerations	Active Tuberculosis	Latent Tuberculosis	Ref.
Diagnostic tools	Interferon Gamma Release Assay blood test (IGRA) Mantoux tuberculin skin test (TST). Patient displays TB symptoms.	Patients display no symptoms of TB with positive IGRA and TST. Further testing such as chest radiograph, and microbiological investigation of sputum.	[19,20]
Standard treatment	Four-month regimen: Intensive phase of a high daily dose of RPT and MOX. INH, PZA for 8 weeks. Continuation phase of RPT, MOX, and INH for 9 weeks. Six-month regimen: Intensive phase chemotherapy of INH, RIF, PZA, and EMB. This regimen varies in frequency of dosing, and lasts 8 weeks. Continuation phase of INH and RIF with different frequency in dosing for 18 weeks/40 weeks for central nervous system TB.	Short course regimens: Three months: INH plus RPT once weekly. Four months: RIF OD. Three months: INH plus RIF OD. Long monotherapy course: 6–9 months of INH monotherapy.	[19,20]

**Table 3:** SAR analysis of the PBTZ class, comparing alkyl groups and effectiveness in treating TB in vivo, and showing that hydrophilic groups lead to loss of antimicrobial activity [156].

Compound #	Alkyl Substitute	MIC Mtb (ng/mL)	LogP
10926013	Methyl	250	1.31
10926021	Ethyl	62	1.64
10926027	Propyl	3.7	2.11
10926172	Butyl	1.9	2.51
11026100	Isobutyl	1.9	2.51
11026142	1-ethylpropyl	0.37	2.99
11026128	1-sec-Butyl	0.37	2.52
11026129	2-Cyclohexylethyl	0.19	3.52
11026131	1-Methylbutyl	0.19	3.11
11026134	Heptyl	0.19	3.30
11026137	4-Phenoxybutyl	1.5	3.35
11026139	4-Phenylbutyl	0.37	4.05
10926168	Cyclohexyl	0.75	3.09
10926169 (PBTZ169)	Cyclohexylmethyl	0.19	3.20
